# Nutrition Knowledge of Dancers: A Scoping Review

**DOI:** 10.3390/nu18152461

**Published:** 2026-07-28

**Authors:** Caitie J. Minger, Matthew B. Cooke, Regina Belski

**Affiliations:** Sport, Performance and Nutrition Research Group, Department of Sport, Exercise and Nutrition Sciences, La Trobe University, Melbourne 3086, Australiamatt.cooke@latrobe.edu.au (M.B.C.)

**Keywords:** dance, nutrition, knowledge, diet

## Abstract

Background/Objectives: Dance is a unique and physically demanding athletic art form that necessitates optimal nutritional intake for performance and long-term health. Nutrition knowledge (NK) is an essential factor that drives dietary behaviours and attitudes of athletes and performers, including dancers. Despite its importance, there appears to be limited research investigating the NK of dancers. This scoping review therefore aims to systematically explore the currently published literature on the NK of dancers globally, to understand the current levels of nutrition knowledge and gaps in knowledge as well as related dietary behaviours and inform future research and resource development for this population. Methods: Four electronic databases (MEDLINE, CINAHL, PubMed, and Web of Science) were searched in June 2026 for articles. Eligibility criteria included: Original research published in peer-reviewed journals between 2000–2025. Only English language studies reporting on the NK (general, sports, specific) of dancers were included. Preferred Reporting Items for Systematic reviews and Meta-Analyses extension for Scoping Reviews (PRISMA-ScR) was followed. Results: Of the 58 studies initially identified, a total of nine studies were included. All were quantitative and either cross-sectional (*n* = 5) or intervention (*n* = 4) studies. Data was collated from six countries with a total of 942 participants, and NK was directly assessed in all of the reviewed papers. The methodological quality and bias were assessed using the “Academy of Nutrition and Dietetics Quality Criteria Checklist for Primary Research” tool. Study designs and tools used to assess NK were heterogenous. Overall, dancers demonstrated low-to-moderate NK, with gaps in knowledge surrounding macronutrient composition of foods, and exhibited restrictive dietary patterns and body image concerns. Reliance on non-expert information sources was prevalent. Interventions designed to improve NK and related dietary behaviours showed positive impacts. Conclusions: Dancers’ NK is low-to-moderate, with suboptimal dietary behaviours, often driven by aesthetic pressures and a reliance on informal nutrition advice. Given the success of interventions to improve NK, future research should focus on targeted nutrition education strategies, focused on identified NK gaps for optimising dancers’ health, performance, and long-term well-being.

## 1. Introduction

Dance is a unique and multifaceted athletic art form encompassing a variety of genres, all of which have their own distinct training and performing demands [1]. Dance, like any physical activity, requires adequate nutrition to meet the energy and physical demands of the exercise, especially for those undergoing intense training and competition schedules. Pre-professional and professional dancers can train/perform for more than 25 h per week, with some training up to 40 h per week [2]. Like other forms of physical activity, this can expend significant amounts of energy, and without sufficient dietary intake of energy and nutrients, may lead to negative health and performance impacts [1]. Dancers can therefore find themselves at a higher risk of energy deficiency and low energy availability (LEA), defined as an individual’s imbalance between energy intake and energy expenditure due to exercise [3]. This risk can be further exacerbated by greater incidences of disordered eating behaviours and eating disorders among dancers, where pressure to maintain a lean physique and still meet their nutritional demands for training and performance is high [4]. While short-term energy deficits can cause fatigue and poor recovery, chronic or problematic LEA, lasting weeks to months, can lead to Relative Energy Deficiency in Sport (RED-S) [3] or Relative Energy Deficiency in Dance (RED-D) [5] syndrome where symptoms such as bone loss, menstrual irregularities, low hormone levels, and weakened immunity become prevalent [3,4]. If left untreated, RED-S can increase injury risk and impair athletic performance [3,4].

Nutrition knowledge (NK) and subsequent dietary behaviours underpin the success of any dietary regime. Both are important for dancers to make informed choices about food, not only to fuel their bodies appropriately for athletic performance but also to support their long-term health, psychological well-being, and the application of nutrition in practice [4,6]. Given the range of energy and physical demands within dance genres, NK is essential for dancers to understand how to fuel their unique nutritional requirements and for health professionals to identify specific gaps to target [4]. To date, very little is known about the NK and related dietary behaviours of dancers. An early study found that pre-professional dancers displayed limited and varied NK, with mixed beliefs about foods and nutrients impacting performance [7]. In another study, while dancers showcased moderate NK, they displayed significant confusion on numerous topics, including macronutrient content of foods, with more than a third incorrectly identifying pasta as a high-fat food [8]. Emerging research supports earlier findings that dancers often showcase limited NK and demonstrate a high reliance on non-expert sources for advice. Additionally, reports of restrictive eating patterns such as meal skipping have been noted alongside elevated risks of disordered eating habits, including use of weight control products (e.g., laxatives and diet pills) [9,10].

Given the relationship between NK and dietary behaviours, including disordered eating, and its impact on dancers’ health and performance, it is evident that research is needed to understand the current landscape of the NK and related dietary behaviours of dancers from all genres to better inform future studies and strategies [4]. This scoping review therefore aims to systematically explore the currently published literature on the NK of dancers globally, to understand the current levels of nutrition knowledge and gaps in knowledge as well as related dietary behaviours and to provide a comprehensive review that can inform future research. This, in turn, can inform the development of NK resources and contribute to the long-term well-being of dancers.

## 2. Materials and Methods

This review was conducted following the Preferred Reporting Items for Systematic reviews and Meta-Analyses extension for Scoping Reviews (PRISMA-ScR) guidelines [11]. This review was not registered, and a protocol was not prepared. Papers published between 2000 and 2025 were included as the interest of the review was the last 25 years of dance data.

### 2.1. Search Terms

A systematic search was conducted (30 June 2026) across MEDLINE, CINAHL, PubMed, and Web of Science. Key search terms (“nutrition”, “knowledge”, dance*) and a complementing Boolean operator (“AND”) were used in a title and abstract search.

### 2.2. Eligibility Criteria

Original research (cross-sectional, observational, intervention) published in peer-reviewed journals between 2000 and 2025 was included. Abstracts, conference posters, and unpublished dissertations and theses were excluded. All levels, experience and types of dance were accepted. Only English language studies reporting on NK (general, sports specific) were included. Intervention studies were not required to include baseline nutrition knowledge outcomes for inclusion; however, they did need to examine and discuss nutrition knowledge in the manuscript.

### 2.3. Selection Process

**Figure 1:**
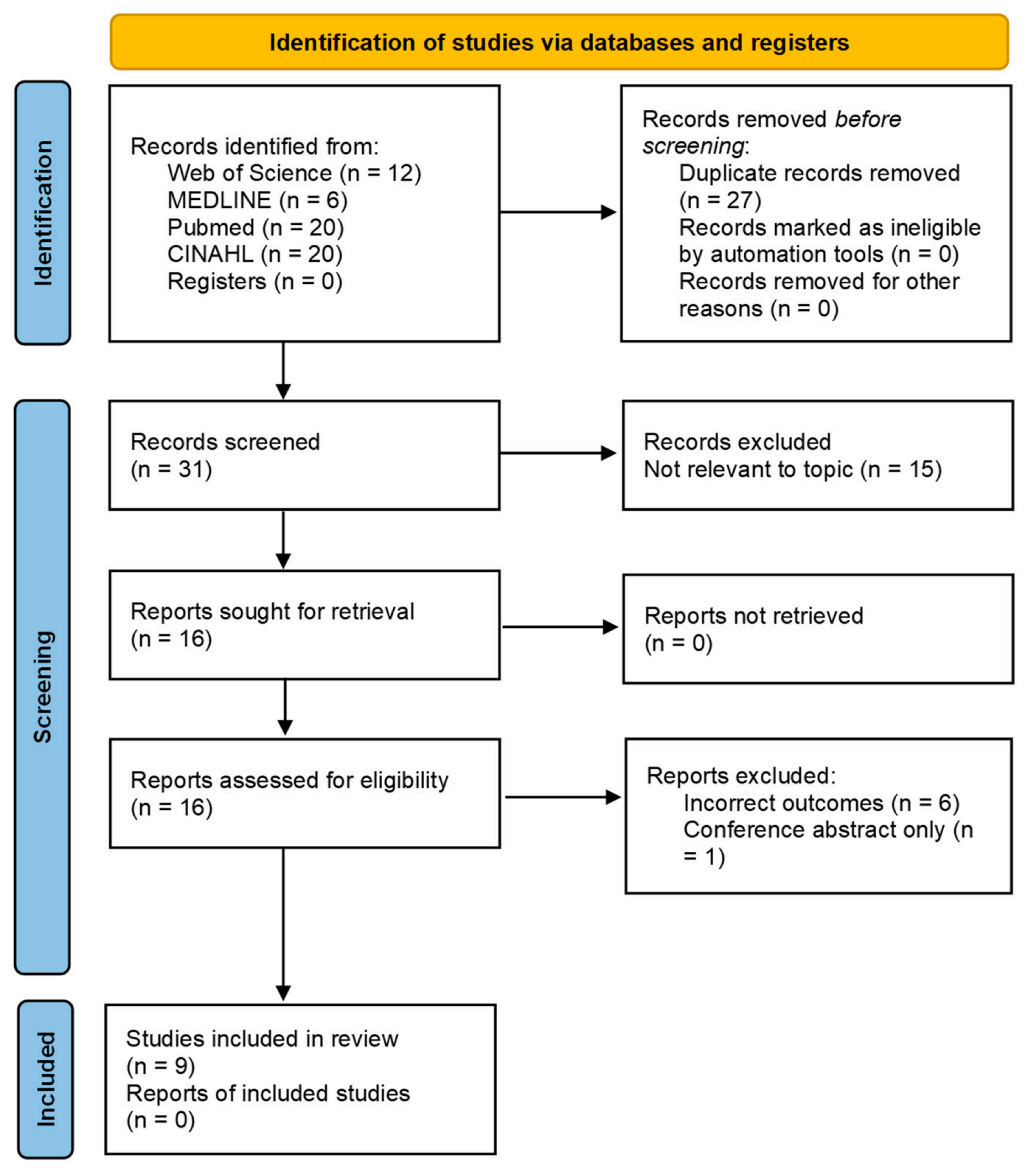
PRISMA flow diagram of the study selection process.

Duplicate articles returned from the search were removed (C.J.M.) and analysed via Covidence [12]. Two reviewers (C.J.M. and R.B.) independently evaluated the titles and abstracts of all retrieved citations for eligibility. The same reviewers then assessed the full texts of relevant articles for inclusion based on the established inclusion/exclusion criteria. Any disagreements were to be resolved by a third reviewer (M.B.C.); however, there were no disagreements in the selection process between the two researchers which required resolution. Figure 1 depicts the PRISMA flow diagram for this scoping review.

### 2.4. Data Extraction

Two reviewers (C.J.M. and R.B.) independently extracted data into Excel from Covidence. Extracted data from all studies included author(s), publication year, country, study design, participant sample characteristics (size, mean age, and gender when available), and key findings. No assumptions were made for missing or unclear data.

### 2.5. Study Quality

The methodological quality and risk of bias of the studies was assessed by two reviewers (C.J.M. and R.B.) using the “Academy of Nutrition and Dietetics Quality Criteria Checklist for Primary Research” tool, being evaluated across 10 criterion and given an “Overall” quality rating according to published cut-off ranges [13]. Any disagreements were to be resolved by a third reviewer (M.B.C.); however, there were no disagreements between the two researchers which required resolution.

## 3. Results

### 3.1. Study Selection

A total of 58 studies were retrieved through electronic database search (Web of Science = 12, Medline = 6, PubMed = 20, CINAHL = 20). After removing duplicate copies, 31 papers remained. Following title and abstract screening, 16 papers underwent a full-text review. Following the full text review, nine papers met the inclusion criteria and were included in the final manuscript version.

### 3.2. Study Quality

**Figure 2:**
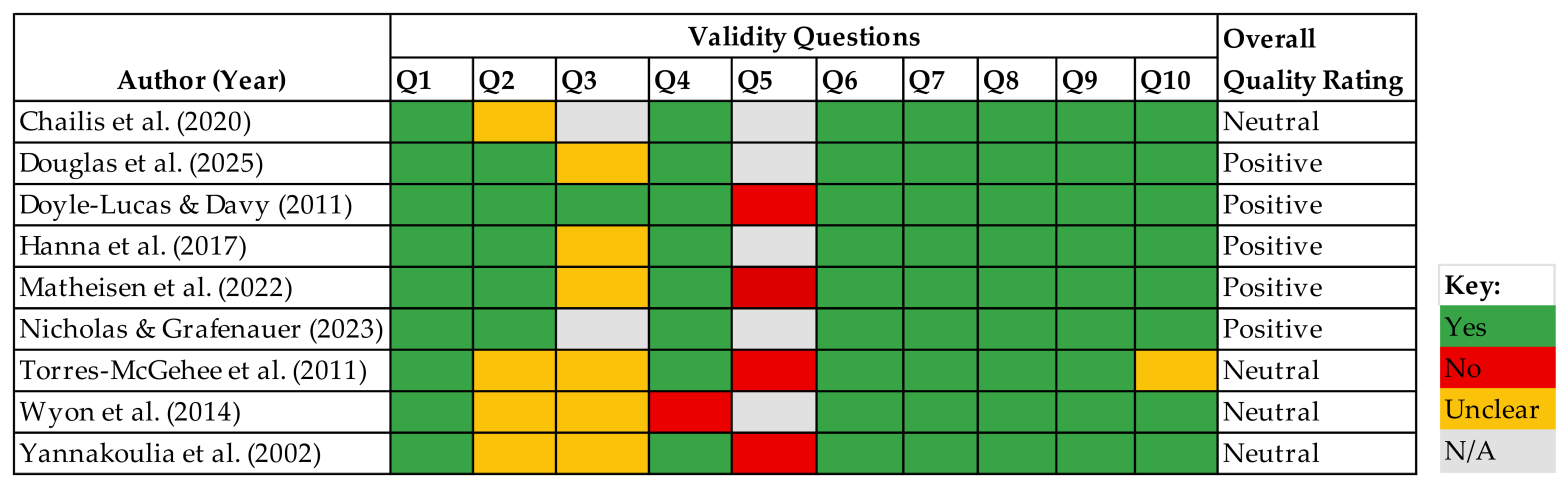
Study quality checklist [1,8,9,14,15,16,17,18,19].

Five out of nine papers received a “positive” study quality rating, with four studies receiving a “neutral” rating (please see summary in Figure 2). A positive rating indicates that the research clearly addresses factors such as bias, generalisability and the methodology. Alternatively, a neutral rating signifies the research is neither strong nor weak in those areas [13]. The studies which received a neutral rating lacked clarity in relation to selection bias (Q2), and/or whether the study groups were comparable (Q3).

### 3.3. Study Characteristics and Nutrition Knowledge Scores

All of the included papers were quantitative and were published between 2002 and 2025. Study designs included cross-sectional (*n* = 5) and intervention (*n* = 4), (please see summary in Table 1). Studies were conducted across six different countries/regions globally. Within the studies, participants were predominantly pre-professional and student dancers, with an average age range of 15–28 years.

### 3.4. Nutrition Knowledge

Whilst all nine studies reported assessing NK, only seven of the nine papers explicitly described NK scores amongst dancers [8,9,14,15,16,17,18], which consistently exposed deficits in general and sports-specific NK; these are summarised in Table 1. Mean NK scores in cross sectional studies and at the baseline of intervention studies reported ranges from 27.3 to 65.2%, with a variety of tools used to assess NK.

Eight different NK assessment tools were used across the nine studies. Three studies used validated tools [8,15,17], three used unvalidated tools [1,18,19], two reported limited piloting or validation [14,16] and another reported significant modification of a previously validated tool, limiting its original validity [9]. Two of the studies [15,17] used different versions of the validated General Nutrition Knowledge Questionnaire (GNKQ) (original) [20] and GNKQ (Australian) [21]. Details of the tools used are presented in Table 1. The heterogeneity of the tools described above needs to be considered in the interpretation of any results.

#### 3.4.1. Cross Sectional Studies

Cross-sectional studies from across the world (*n* = 5) were relatively consistent in reporting mean NK levels. A recent study by Douglas et al. [9] of US collegiate dancers demonstrated low baseline proficiency, achieving an average score of just 43.4% on a modified Nutrition Knowledge Questionnaire (NKQ) [22], with only 12% of the entire cohort scoring above 60%. Similarly, Challis et al. [8] reported a moderate NK score of 47% amongst professional or fulltime Irish dancers using a previously validated tool, The Sport Nutrition Questionnaire. This was also consistent in an Australian cohort of pre-professional dancers, and dance degree students surveyed by Hanna et al. [15] who observed low NK, with 47.3% and 52.2% of responses correct in dance and dance performance students, respectively. Interestingly they did not observe a difference in NK levels between dancers at first or later years of university [15]. Wyon et al. [17] examined the association between eating behaviours, BMI, and NK amongst elite student dancers and professional ballet dancers in the UK. The study involved dancers completing the previously validated GNKQ, revealing that mean NK scores varied by age group, from 27.3 to 49.1%, with knowledge scores positively correlating with age. The findings of this study also revealed that professional dancers possessed significantly higher NK alongside a higher BMI compared to student dancers [17]. Whilst Nichloas and Grafenauer [1] reported assessing NK, insufficient details were provided in their manuscript to determine a NK score.

#### 3.4.2. Intervention Studies

Intervention studies (*n* = 4) included in the review included interventions ranging from those taking place over a period of 3 consecutive days up to 3 months, predominantly within pre-professional collegiate dancers [14,16,18,19]. Three out of the four intervention studies also did a follow-up survey ranging from 6 weeks [14] to 6 months [18,19].

**Table 1:** Study characteristics.

Author, Year	Country/ Region	Age (Years)	Sample Size/Gender	Genre	Population	Study Design	NK Tool Used	Validation Status	NK Scores
Intervention Studies									
Yannakoulia et al. [18] 2002	Greece	Range: 19–25 20.5 ± 1.6 (Mean ± SD)	Total *n* = 32 F	N/A	Pre-professional (dance school) dancers	Quantitative, intervention 12 weeks duration, 1 × 2 h nutrition education session per week, (prevention of eating disorder and applied nutrition for dancers). 6-month follow-up Other Nutrition Related Measures: EAT-26, DEBQ, BDQ, Weight Dissatisfaction Scale	Nutrition Knowledge Test	Unvalidated	Baseline: 59.2% Post Intervention: 94.8%
Torres-McGehee et al. [16], 2011	USA	Range: 18–25 Intervention 19.2 ± 1.2 (Mean ± SD) Control 19.1 ± 1.0 (Mean ± SD)	Total *n* = 40 F Intervention *n* = 21 F Control *n* = 19 F	Auxiliary dancers	Pre-professional (college) dancers (Having to be a member of a dance unit for at least 1 year)	Quantitative, intervention 4 weeks duration, 2 × 45-min sessions per week (team-centred program on sport nutrition, exercise, and disordered eating consequences). Other Nutrition Related Measures: EDI-3, CES-D, Eating Disorder Knowledge Questionnaire	Nutritional Knowledge Survey [23]	Partial Validation	Baseline: Control 43.2% Intervention 46.4% Post Intervention: Control 44.7% Intervention 71.4%
Doyle-Lucas & Davy [14], 2011	USA	Range: 13–18 Intervention 15.4 ± 0.1 (Mean ± SEM) Control 15.4 ± 0.1 (Mean ± SEM)	Total *n* = 321 Intervention *n* = 231 217 F, 14 M Control *n* = 90 84 F, 6 M	Ballet	Pre-Professional dancers (affiliated with professional dance companies)	Quantitative, intervention 3 × 10–20 min nutritional education intervention through a DVD lecture series over 3 consecutive days. 6-week follow-up Other Nutrition Related Measures: Nutrition education program data	SNKBQ Investigator-designed	Piloted, Unvalidated	Baseline: Intervention 65.2% Control 62.9% Post Intervention: Intervention 93.4% 6 weeks post-follow-up Intervention 91.0%
Mathisen et al. [19], 2022	Norway	Intervention Mean 20.7 (CI 20.3–21.1) Control Mean 28.7 (CI 25.1–32.3)	Intervention *n* = 125 111 F, 14 M Reference *n* = 39 33 F, 6 M	Jazz (*n* = 72) Contemporary (*n* = 53)	Pre-professional dancers (dance students)	Quantitative, intervention 3 months duration, 1 × 90-min workshops per month (on nutrition, eating disorder and mental health) over 3 consecutive months. 6-month follow-up Other Nutrition Related Measures: Body Appreciation Scale, Hopkins Symptom Check list, Eating Disorder Examination Questionnaire, LEAF-Q, LEAM-Q	NK assessed using investigator-designed tool	Unvalidated	Insufficient details provided on NK tool or scores to determine % knowledge.
Cross-Sectional Studies									
Wyon et al. [17], 2014	United Kingdom	Range: 11–39	*n* = 180 111 F, 69 M	Ballet	Pre-professional (dance school, 4–6 h of ballet training 6 days/week) & professional dancers (contracted for 38 h of dance/week)	Quantitative, cross-sectional Other Nutrition Related Measures: EAT-26	GNKQ [20]	Validated	Mean GNKQ scores varied depending on age group, 27.3–49.1%.
Hanna et al. [15], 2017	Australia	Ranges: <18 (*n* = 12) 18–24 (*n* = 55)	*n* = 67 65 F, 2 M	Ballet, contemporary, alternative, body-conditioning dance styles	Pre-professional dancers, and dance degree students (one university, all levels), convenience sample	Quantitative, cross-sectional Other Nutrition Related Measures: Active Australia Survey	GNKQ-Australian [21] (Validated)	Validated	Dance students: 47.3% and Dance Performance students: 52.2%
Challis et al. [8], 2020	Ireland	F 21 ± 3 (Mean ± SD) M 27 ± 8 (Mean ± SD)	*n* = 40 35 F, 5 M	Irish dance	Pre-professional (full time training) & professional dancers	Quantitative, cross-sectional Other Nutrition Related Measures: EAT-26, Food Diaries	The Sport Nutrition Questionnaire [24]	Validated	47%
Douglas et al. [9], 2025	USA	20.8 ± 2.5 (Mean ± SD)	*n* = 35 31 F, 4 M	Ballet (*n* = 5) Modern (*n* = 24) Jazz (*n* = 2) Other (*n* = 4)	Pre-professional dancers (college, all levels), Minimum 15 h dance per week	Quantitative, cross-sectional Other Nutrition Related Measures: EAT-26, BIAS-BD	Adapted version of NKQ [22]	Modified	43.4% (only 12% obtained over 60%)
Nicholas & Grafenauer [1], 2023	Australia	Range: 16–24 19.2 ± 1.4 (Mean ± SD)	*n* = 63 56 F, 4 M, 3 Other	Classical ballet, Contemporary/modern, Jazz/musical theatre, Hip hop/urban/commercial, Tap	Pre-professional dancers (dance academy, enrolled in full-time dance program)	Quantitative, cross-sectional Other Nutrition Related Measures: DEAQ	NK assessed as part of investigator designed survey	Unvalidated	Insufficient details provided on NK tool or scores to determine % knowledge.

Table Abbreviations: SD: Standard Deviation, SEM: Standard Error of the Mean, CI: Confidence Interval, NK: Nutrition Knowledge, M: Male, F: Female, SNKBQ: Sports Nutrition Knowledge and Behaviour Questionnaire, GNKQ: General Nutrition Knowledge Questionnaire, NKQ: Nutrition Knowledge Questionnaire, EAT-26: Eating Attitudes Test, DEBQ: Dutch Eating Behavior Questionnaire, BDQ: Body Part Dissatisfaction Questionnaire, EDI-3: Eating Disorder Inventory, CES-D: The Center for Epidemiological Studies Depression Scale, LEAF-Q: Low Energy Availability in Females Questionnaire, LEAM-Q: Low Energy Availability in Males Questionnaire, BIAS-BD: Body Image Assessment Scale-Body Dimensions tool, DEAQ: Dance-Specific Energy Availability Questionnaire.

Yannakoulia et al. [18] utilised a 12-week intervention, which was inclusive of two-hour education sessions every week for twelve weeks, in Greek dancers, demonstrating a marked improvement in NK scores (mean scores before and after intervention: 59.2% and 94.8%, respectively), with this improvement largely maintained at 6-month follow-up (NK 90%). Torres-McGhee et al. [16] conducted a much shorter intervention, over 4-weeks, with weekly education sessions on sports nutrition and disordered eating risk for US auxiliary dancers. Dancers who attended the weekly education sessions significantly improved their NK scores compared to the control group, who received no education, with mean scores improving from a pre-test baseline of 46.4% to a post-test mean of 71.4% in the intervention group. In contrast, the control group showed minimal change [16]. Taking a less intensive approach, utilizing technology, Doyle-Lucas & Davy [14] implemented a theoretically based nutritional education program through a DVD lecture series (three 10–20 min classes) over three consecutive days, during a summer intensive program for pre-professional, adolescent ballet dancers in the US. Significant improvements in NK were observed in the intervention group from baseline to immediate post-program assessments from 66% to 93% correct. This was also largely maintained at the 6-week follow-up (NK 91%) [14]. In another study, Mathisen et al. [19] implemented three 90 min workshops covering: nutrition, eating disorders and mental health, held during school hours, for one session per month over three months, for pre-professional Jazz and contemporary dancers in Norway. They reported that the intervention was successful at increasing the nutritional knowledge among dancers, with small to moderate effects and with no significant difference from the reference group, unfortunately insufficient details were provided on the NK tool or actual scores to determine baseline NK or percentage change [19].

#### 3.4.3. Nutrition Knowledge Gaps

Specific gaps in NK were also highlighted in several of the studies. Nutrition knowledge was consistently found to be inadequate, particularly in relation to energy and macronutrient needs, and content of foods, as well as sports nutrition principles and impact of under fuelling on health and diet–disease relationships [1,8,9,14,15,16,17,18,19]. For example, Challis et al. [8] observed gaps in knowledge relating to macronutrient content of foods, whereby the mean score for correctly identifying foods high or low in carbohydrates was 68%, for foods high or low in protein 78%, and for foods high or low in fat only 39%. With 36% of participants wrongly identifying pasta as a high-fat food and 28% identifying butter as high in carbohydrates. A couple of the papers also reported on differences by age/experience [15,17]. Wyon et al. [17] demonstrated that professional dancers had significantly greater food knowledge than the student dancers. Analysis by student year group in addition to the professional dancer group indicated a significant increase by year with no gender differences. To the contrary, Hanna et al. [15] observed that nutrition knowledge did not vary between students in first or later years in a group of Australian university dance students.

### 3.5. Additional Findings on Nutrition Information Sources and Related Dietary Behaviours

#### 3.5.1. Nutrition Information Sources

Two studies included details on the source of nutrition information and noted that dancers frequently relied on informal and potentially unreliable sources [1,9]. With Douglas et al. [9] reporting that dancers primarily sourced nutrition information from peers (65.7%), family (60%), friends (57.1%) and coaches/trainers (37.1%). Similarly, Nicholas and Grafenauer [1] highlighted that a significant proportion of dancers reported obtaining advice from internet sources (42.2%) and friends (28.1%), while 24% of participants reported feeling pressured by teachers to exclude particular foods in the past. Additionally, when Nicholas and Grafenauer [1] asked about the impact of social media, 81.3% of dancers reported that social media makes them feel like they should “sometimes” or “always” try and lose weight. It was not clear whether reliance on peers, family, and social media differed by age, experience level, or dance genre.

#### 3.5.2. Related Dietary Behaviours

Dietary behaviours and attitudes were reported in the majority of the included studies, demonstrating that nutritional behaviours and attitudes amongst the dancing community are often characterised by restrictive patterns and a drive for maintaining a lean physique, supporting earlier reports in the literature [1,8,9,14,16,17,18,19]. Several studies showed that low nutrition knowledge often coexisted with disordered eating behaviours, body image concerns and inadequate dietary intake [14,16,17,18,19]. Douglas et al. [9] highlighted that some participants were at risk of an eating disorder and of these, 33% used laxatives and 50% used diet pills. Moreover, Nicholas and Grafenauer [1] noted that 44% of participants reported excluding food groups in their diet.

## 4. Discussion

This scoping review systematically explored the currently published literature on NK and of dancers globally to identify key themes and gaps in the literature. However, it was limited to only those papers published in English, limiting the overall scope. Nine papers met the inclusion criteria and were included in the review. Generally, there was a range of literature from across the world alongside a range of dance genres and levels, which provided valuable insight into the variability of NK amongst dancers. Overall, NK scores were low-to-moderate amongst dancers, regardless of genre and experience. Significant gaps in understanding of macronutrient requirements were identified. A potential contributing factor may be the reported reliance on information from informal sources which was highlighted in two of the papers [1,9]; however, this hypothesis is based on limited evidence rather than a consistent finding across the literature and requires further exploration. Reviewed studies employing nutrition educational training or interventions demonstrated improvements in NK amongst dancers [14,16,18,19].

### 4.1. Nutrition Knowledge

The major findings of this review align with previous research, with many studies reporting low to moderate NK (mean levels 27.3 to 65.2%) amongst dancers globally, using a range of heterogeneous NK tools, and across varied dance genres and ages [1,8,9,14,15,16,17,18,19].

Beyond overall NK scores, studies included in this review have consistently demonstrated gaps in dancers’ knowledge in regards to specific areas, including macronutrients and diet–disease relationships [8,15]. Challis et al. [8] demonstrated that over a third of participants identified pasta as a high-fat food. Similarly, more than a quarter of participants wrongly thought that butter was a food item high in carbohydrates. Whilst research by Hanna et al. [15] showed that dancers scored better on questions focussed on dietary recommendations and lowest in diet–disease relationship.

When distinguishing knowledge levels across age groups or years of experience, results were inconsistent. Whilst some researchers found that professional dancers had significantly greater NK compared to student dancers and that NK increased with age [17], others observed no difference in NK scores between dance students in first or later years of university [15]. It is important to note that the age range may have been too narrow in the latter study to observe a statistically significant difference. Previous education, specifically nutrition education/courses was an influencing factor with individuals previously engaged in such courses demonstrating slightly higher scores [9], and all four intervention studies included showed an increase in NK post education interventions, regardless of length or style of intervention [14,16,18,19].

The heterogeneity of the assessment tools used in the reviewed literature limited the ability to compare these findings accurately and necessitates caution in interpreting NK scores and the coinciding validity of results. Clearly, there is a need for further research to be conducted using consistent validated tools known to be reliable and accurate in measuring NK within athletic populations, using tools such as the Abridged Nutrition for Sport Knowledge Questionnaire (ANSKQ) [25], which has had extensive validation, and has been translated widely, should be considered. The findings from this scoping review clearly highlight significant gaps in NK among dancers. It demonstrates that on average, dancers possess a low–moderate level of NK, with most scoring well below 60–70%. Specifically, there are apparent limitations in understanding the role of macro- and micronutrients in the diet and in accurately identifying macronutrient content in selected foods, which proves to be a concern for what dancers are consuming [8].

### 4.2. Related Dietary Behaviours

Dietary behaviours and attitudes were reported in most of the included studies, demonstrating that nutritional behaviours and attitudes amongst the dancing community are often characterised by restrictive patterns and a drive for maintaining a lean physique [1,9,14,16,17,18,19]. Several studies showed that low nutrition knowledge often coexisted with disordered eating behaviours, body image concerns and inadequate dietary intake, highlighting the need for targeted, dance-specific nutrition education delivered by qualified professionals [1,9,16,18]. This is supported by other literature, with a recent cross-sectional study by Batista et al. [6] highlighting how dancers emphasised the importance of a lean physique for their career alongside using methods such as dieting, fasting and calorie restricting, contributing to body dissatisfaction, anxiety and depression, all being detrimental to their athletic ability and health. Dancers have also been shown to express an increased desire to limit their intake and types of food before performance or rehearsal, which they believed could lead to increased fatigue and decreased performance, viewing post-performance intake as a reward rather than fuel [10].

Intervention studies in this review showed promise in addressing these behaviours, Yannakoulia et al. [18] observed significant improvement in eating behaviours post nutrition questionnaire and weekly education workshops. They found an additional reduction in abnormal eating behaviours, which was maintained 6 months post-intervention. However, they noted a slight decline in NK scores at follow-up. Interestingly, participants at high risk of abnormal eating behaviours benefited the most [18].

The identified knowledge deficits are of particular concern when coupled with negative or skewed dietary behaviours and attitudes towards food. Since the early 2000s, the literature has showcased a deficit in nutritional behaviours, with authors highlighting a significant prevalence of meal skipping and food restricting due to the perceived impact on performance, as well as concerns about dietary deviations and binge eating habits [10]. In the current review, research by Nicholas and Grafenauer [1] also highlighted that body image concerns were exacerbated by negative commentary from friends and family members and the majority of dancers felt that social media contributed to the feeling of having to lose weight and over half of these indicated that diet-restricting habits were affecting them. Therefore, there is a need to further explore whether there is indeed a link between social media and access to information and perceived pressure from non-experts or unreliable sources and negative food-body relationship of dancers [6]. Finally, the previously reported favourable view held by dancers in regards to being of a lighter weight, as well as the associated reporting of past/current difficulties with food–body relationships, demonstrates that retention and application of NK and practices in their life may be influenced by other factors, and that warrants additional research in the field [9]. It is important to highlight that some of the described behaviour may fit the definition of disordered eating, which encompasses subclinical maladaptive eating behaviours and attitudes, which is distinct from clinical eating disorders which are formally recognised psychiatric conditions characterised by persistent disturbances that meet established diagnostic criteria [26]. Both may have a negative impact on dancers, and hence it may be wise for researchers and dance staff to also consider screening dancers for disordered eating risk behaviours to enable early intervention. Suitable, simple, validated tools now exist to be able to do so, including the Athletic Disordered Eating (ADE) Screening Tool [26].

### 4.3. Limitations

It is important to re-emphasise that the interpretation of findings from this review is limited by substantial heterogeneity across the included studies. Variability in sample characteristics, including differences in age, training level, and professional status, alongside representation from multiple countries and dancer populations, reduces comparability and generalisability of results. In addition, the inclusion of diverse dance genres with distinct physiological and cultural demands further complicates the synthesis of findings, as nutritional requirements and behaviours may differ considerably between disciplines. Importantly, the use of a wide range of NK assessment tools, some validated and others with unclear validity, limits the reliability and consistency of reported outcomes and restricts meaningful comparison across studies. Collectively, this heterogeneity necessitates cautious interpretation of results and highlights the need for more standardised methodologies in future research. Additionally, papers in languages other than English were also excluded and this is a limitation of the review.

### 4.4. Future Directions

Despite the aforementioned limitations, and the wide variety of methodological approaches used by researchers to assess NK, the data consistently shows low-to-moderate NK levels, and that nutrition education interventions have the positive effects of: increasing NK, positive changes in eating behaviours, and a decrease in concerning eating patterns of dancers [14,16,18,19]. This is further supported by the findings from the literature that engaging in prior nutrition education or courses seems to directly correlate with higher NK scores among dancers [9]. Moreover, the data revealed that professional dancers may have higher NK scores compared to pre-professional dancers [17]; this may relate to more opportunistic exposure to nutrition education during their development. As a result, it is vital for future researchers to target student and pre-professional dance populations with the most effective intervention methods. It is critical that staff working with dancers consider their NK gaps. They should focus on using validated NK tools (e.g., ANSKQ [25]) to assess current NK levels and gaps in knowledge of the dancers they are supporting. This can then inform the development of targeted nutrition education resources and practical strategies to target key nutrition development areas as well as improve dancers’ relationship with food and body and understanding of the role and importance of appropriate nutrition in respect to both their performance and health as a dancer. These should be fit-for purpose and sustainable intervention programs to improve NK over time. Additionally, where possible, interventions should be delivered by qualified dietitians/sports dietitians, given the identified reliance on non-expert information sources, and the negative impact erroneous information may cause dancers.

## 5. Conclusions

This review has identified that dancers’ NK is low-to-moderate regardless of dance genre, level or age. Dancers also appear to rely on non-expert sources for nutrition advice. Related dietary behaviours amongst dancers appear to be characterised by restrictive patterns and a drive for a lean physique, which may contribute to suboptimal dietary intake and performance outcomes. Interventions focused on improving NK and dietary behaviours showed a positive impact; however, only a limited number of intervention studies were identified and the intervention methodologies varied substantially. This review highlights that there is room for improvement in the NK and dietary behaviours of dancers. Effective nutrition-related interventions that focus on optimising dancers’ NK and related dietary behaviours should be considered, as they may positively impact dancers’ overall health and dance performance. Future research should also investigate sources of nutrition information used by dancers and use validated tools to assess both NK and food–body relationships of dancers.

## Data Availability

No new data were created or analysed in this study. Data sharing is not applicable to this article.

## Abbreviations

The following abbreviations are used in this manuscript:

NK	Nutrition Knowledge
EI	Energy Intake
LEA	Low Energy Availability
RED-S	Relative Energy Deficiency in Sport syndrome
RED-D	Relative Energy Deficiency in Dance syndrome
ANSKQ	Abridged Nutrition for Sport Knowledge Questionnaire
ADE	The Athletic Disordered Eating Screening Tool
GNKQ	General Nutrition Knowledge Questionnaire
NKQ	Nutrition Knowledge Questionnaire
SNKBQ	Sports Nutrition Knowledge and Behaviour Questionnaire
